# Post-marketing safety evaluation of anthracycline for acute myeloid leukemia treatment: a real-world pharmacovigilance analysis

**DOI:** 10.3389/fphar.2026.1759201

**Published:** 2026-06-10

**Authors:** Yong Wang, Qian Liu, Jing Xu, Fei Yang, Meiyan Wang, Haoxiang Lu, Jie Wu, Ao Zhang, Xingli Zhang, Dehong Wu, Hongchun Qiu

**Affiliations:** Department of Hematology and Oncology, Kunshan Third People’s Hospital, Suzhou, China

**Keywords:** acute myeloid leukemia, anthracyclines, drug safety, FAERS, pharmacovigilance

## Abstract

**Background:**

Anthracyclines are key for acute myeloid leukemia (AML) but carry serious side effects. This study identifies their side effects across drugs and patient populations using the FDA Adverse Event Reporting System (FAERS).

**Materials and methods:**

We analyzed 1,079 AML cases and 3,622 adverse events (AEs) from FAERS (2004–2024). Disproportionality analyses (PRR, ROR, BCPNN, MGPS) detected AE signals; Kruskal–Wallis tests evaluated time-to-onset by age/sex.

**Results:**

Distinct risks emerged: epirubicin (strongest cardiotoxicity, ROR = 10.57), doxorubicin (highest pregnancy-related AEs, ROR = 9.97), daunorubicin (vascular disorders, ROR = 2.38), idarubicin (myelosuppression/infections). Most AEs occurred within 180 days. Idarubicin showed delayed onset in elderly (P < 0.05); males had delayed doxorubicin toxicity (P = 0.04) but accelerated idarubicin onset (P = 0.049) vs. females.

**Conclusion:**

This analysis identifies distinct safety profiles. Epirubicin and doxorubicin were associated with cardiac and pregnancy-related risks, while idarubicin showed signals suggesting potentially delayed onset in elderly patients. Observed sex differences indicate possible variations in AE patterns.

## Introduction

Acute myeloid leukemia (AML), the most common acute leukemia in adults with rising incidence (Schlenk, 2023), is a life-threatening clonal hematopoietic malignancy characterized by disrupted hematopoiesis and bone marrow failure (Venugopal and Sekeres, 2024). As a cornerstone of AML chemotherapy (Bhagat and Kleinerman, 2020), Anthracyclines exert antileukemic effects through ROS/RNS-mediated cellular damage and DNA intercalation with topoisomerase II inhibition (Bhagat and Kleinerman, 2020). Clinical evidence demonstrates that anthracyclines effectively induce tumor cell apoptosis, significantly improve complete remission rates, and create opportunities for subsequent consolidation therapy or hematopoietic stem cell transplantation, making them indispensable in AML treatment (Tuzovic et al., 2018).

Despite their efficacy, anthracycline safety remains a clinical concern. Cardiotoxicity is the most widely studied adverse effect (Mattioli et al., 2023). This includes acute reactions (Monteiro et al., 2021) like arrhythmias and myocarditis (Dazzi et al., 2001), as well as chronic heart damage ranging from subclinical left ventricular ejection fraction decline to heart failure (Neuendorff et al., 2020). A prospective clinical study demonstrated that while both epirubicin and doxorubicin caused similar systolic dysfunction at low doses, only doxorubicin significantly impaired diastolic function (Cottin et al., 1998). Other anthracycline toxicities include alopecia, nausea (Savani et al., 2021), myelosuppression (McGowan et al., 2017), gastrointestinal, and hepatorenal complications (Sheibani et al., 2022). However, most studies only focus on single-agent anthracycline or single toxicity. Only a few preclinical studies have systematically compared toxicity profiles across anthracycline analogs. A golden hamster study revealed that doxorubicin and daunorubicin exhibit the most severe cardiac damage and alopecia, while aclacinomycin and tetrahydropyranyl-adriamycin show minimal toxicity (Dantchev et al., 1984). The toxicity profiles and incidence rates vary across anthracycline types remains unclear.

Emerging evidence highlights substantial variations in toxicity profiles across different patient populations. Older AML patients face elevated risks of anthracycline-related left ventricular dysfunction (ARLVD) due to prevalent cardiac comorbidities, necessitating comprehensive baseline cardiac evaluation and risk stratification (Neuendorff et al., 2020). Similarly, patients with Down syndrome demonstrate increased susceptibility to cardiotoxicity, particularly at standard anthracycline doses, suggesting the need for dose modifications (Hefti and Blanco, 2016). These findings underscore the critical need for population-specific toxicity monitoring for high-risk patients.

Therefore, this post-market drug safety study aims to investigate the side effects of anthracycline drugs in treating AML, as well as the incidence and distribution patterns of adverse reactions across different anthracycline agents and patient populations, using the FDA Adverse Event Reporting System (FAERS) database. The findings are expected to optimize individualized treatment selection and refine AML therapeutic strategies, ultimately improving clinical outcomes for patients.

## Materials and methods

### Data sources and procedures

Pharmacovigilance data were extracted from the FDA Adverse Event Reporting System (FAERS) database (https://www.fda.gov/drugs/questions-and-answers-fdas-adverse-event-reporting-system-faers), covering reports from the first quarter (Q1) of 2004 to the fourth quarter (Q4) of 2024, with focus on post-marketing use of anthracyclines in AML treatment. The FAERS database, compliant with ICH International Safety Reporting Guidelines (ICH E2B), employs MedDRA for adverse event (AE) coding and comprises seven data files: demographic and administrative information (DEMO), drug information (DRUG), adverse event terms (REAC), patient outcomes (OUTC), report sources (RPSR), therapy start/end dates (THER), and drug indications (INDI).

Anthracycline-related reports were identified using generic names, common abbreviations, and trade names as search terms (Hauben et al., 2021). Drugs were categorized by reported role in AEs as primary suspect (PS), secondary suspect (SS), concomitant (C), or interacting (I). AE data were standardized using MedDRA preferred terms (PT) and systematically classified by system organ class (SOC). Data cleaning was performed using SAS statistical software (version 9.4; SAS Institute, Cary, NC, United States).

**FIGURE 1:**
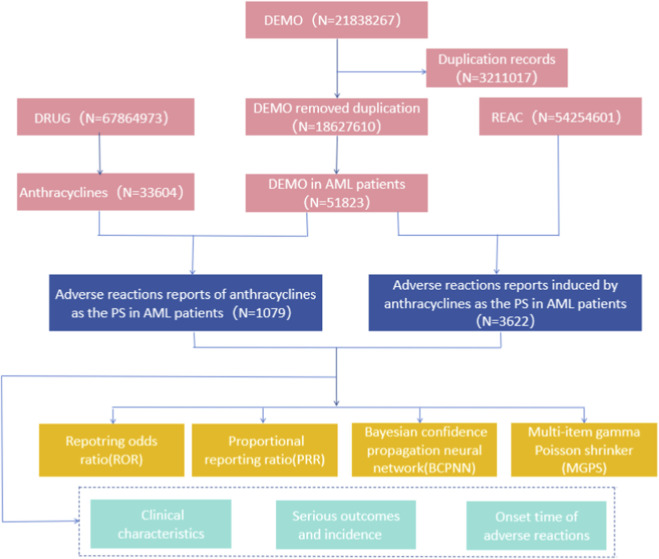
Flowchart of data cleaning process for adverse event data of anthracycline for acute myeloid leukemia based on the FAERS database. FAERS, Food and Drug Administration Adverse Event Reporting System.

As shown in Figure 1, we initially extracted 21,838,267 reports from the DEMO table. After removing 3,211,017 duplicate records based on CASEID, FDA_DT, and PRIMARYID, 18,627,610 unique reports remained. From this deduplicated dataset, we identified 33,604 reports where an anthracycline was listed as the PS drug. Restricting to patients with AML using the INDI files yielded 51,823 AML-related reports. Intersecting these two datasets produced our final analytic sample: 1,079 reports of adverse reactions induced by anthracyclines as the PS drug in AML patients, containing 3,622 associated adverse events. The anthracyclines analyzed were daunorubicin, doxorubicin, epirubicin, and idarubicin.

### Statistical analysis

Disproportionality analysis was conducted using multiple methodologies: frequentist approaches (proportional reporting ratio [PRR] and reporting odds ratio [ROR]) alongside Bayesian methods (Bayesian confidence propagation neural network [BCPNN] and multi-item gamma Poisson shrinker [MGPS]) (Hauben et al., 2021; Lindquist et al., 2000). We employed multiple methods to leverage the complementary strengths of each: PRR and ROR provide simplicity and are suitable for small datasets, whereas BCPNN and MGPS offer robust handling of sparse data and improved detection of true signals (Sakaeda et al., 2013). This multi-method approach allows cross-validation and enhances confidence in identified signals. Given the exploratory nature of disproportionality analyses in pharmacovigilance, formal multiple testing corrections were not applied. Identified signals should therefore be interpreted as hypothesis-generating rather than confirmatory. The signal detection thresholds were selected according to widely accepted conventions in spontaneous reporting system analyses (Dai et al., 2024; Lin et al., 2025). Specifically, a signal for ROR was defined as a lower bound of the 95% confidence interval (CI) greater than 1 with at least three reports, while for PRR, a signal was defined as PRR ≥2 with χ^2^ ≥ 4 and at least three cases, consistent with the criteria originally proposed for signal detection in pharmacovigilance research. For BCPNN, a signal was considered present when the lower limit of the 95% credibility interval (IC025) exceeded 0, in line with recommendations from the WHO Uppsala Monitoring Centre. For MGPS, a signal was defined as EBGM05 >2, following established Bayesian shrinkage methodology. These thresholds are commonly applied in FAERS-based studies to identify statistically disproportionate reporting patterns and are intended for signal detection rather than causal inference. When discrepancies occurred (e.g., ROR/PRR positive but IC025 negative), we interpreted the signal conservatively and required consistency in at least two approaches to classify it as positive. A 2 × 2 contingency table of drug-AE associations is provided in Supplementary Table S1, with detailed formulas and thresholds presented in Supplementary Table S2. The Kruskal–Wallis test was employed to analyze temporal distribution patterns of time-to-onset (TTO) of anthracycline-induced AEs.

Subgroup analyses by age (<18, 18–60, ≥60 years) and sex were performed as exploratory stratifications to partially account for patient heterogeneity. These analyses were conducted separately within each anthracycline agent to assess age- and sex-specific differences in time-to-onset. However, standard disproportionality methods (PRR, ROR, BCPNN, MGPS) are based on 2 × 2 contingency tables and do not support multivariable adjustment for comorbidities, concomitant medications, or reporting year. Therefore, observed differences should be interpreted cautiously and regarded as hypothesis-generating rather than confirmatory. Data processing and visualization were performed using R software (version 4.2.1).

## Results

### Basic clinical characteristics

The demographic and clinical features of anthracycline-associated adverse events (AEs) in acute myeloid leukemia patients are presented in Table 1. A total of 1,079 reports were analyzed, comprising 509 cases for idarubicin, 385 for daunorubicin, 170 for doxorubicin, and 15 for epirubicin. Due to the small sample size for epirubicin, percentages should be interpreted with caution. Idarubicin and daunorubicin were primarily used in middle-aged and elderly patients, while doxorubicin and epirubicin were more common in younger populations. Epirubicin showed the highest proportion of female users (86.7%), with other agents demonstrating balanced gender distribution. Most reports for idarubicin and daunorubicin came from physicians, whereas doxorubicin reports were predominantly submitted by other healthcare professionals (59.4%). The majority originated from the United States, particularly for daunorubicin (55.1%). Clinical outcomes revealed daunorubicin had the highest mortality rate (31.2%), while epirubicin showed the highest hospitalization rate (46.7%). The median time-to-onset for AEs ranged between 6 and 7 days.

**TABLE 1:** Demographic information of anthracycline for treating acute myeloid leukemia.

​	Idarubicin (n = 509)	Daunorubicin (n = 385)	Doxorubicin (n = 170)	Epirubicin (n = 15)
Age (median [IQR])	57.00 [41.00, 65.00]	61.00 [46.00, 68.00]	36.00 [20.25, 55.00]	39.00 [32.00, 69.00]
Sex (%)				
Female	202 (39.7)	189 (49.1)	60 (35.3)	13 (86.7)
Male	239 (47.0)	178 (46.2)	50 (29.4)	1 (6.7)
Missing	68 (13.4)	18 (4.7)	60 (35.3)	1 (6.7)
Year (top 3)				
1	year 2019: 58 (11.4)	year 2004: 65 (16.9)	year 2017: 50 (29.4)	year 2022: 5 (33.3)
2	year 2018: 53 (10.4)	year 2022: 54 (14.0)	year 2018: 22 (12.9)	year 2023: 4 (26.7)
3	year 2020: 45 (8.8)	year 2005: 49 (12.7)	year 2023: 14 (8.2)	year 2019: 3 (20.0)
Reporter’s type of occupation (%)				
Consumer	23 (4.5)	9 (2.3)	5 (2.9)	1 (6.7)
Pharmacist	82 (16.1)	56 (14.5)	34 (20.0)	6 (40.0)
Physician	205 (40.3)	135 (35.1)	29 (17.1)	4 (26.7)
Other health-professional	153 (30.1)	122 (31.7)	101 (59.4)	4 (26.7)
Registered Nurse	26 (5.1)	20 (5.2)	1 (0.6)	0 (0.0)
Missing	20 (3.9)	43 (11.2)	0 (0.0)	0 (0.0)
Reported countries (top 3)				
1	US 153 (30.1)	US 212 (55.1)	Russia 38 (22.4)	France 9 (60.0)
2	France 71 (13.9)	Canada 100 (26.0)	US 28 (16.5)	China 3 (20.0)
3	Germany 52 (10.2)	UK 17 (4.4)	Japan 15 (8.8)	Japan 2 (13.3)
Drug-induced time (median [IQR])	7.00 [6.00, 23.50]	7.00 [4.00, 22.00]	6.50 [2.25, 31.00]	6.00 [6.00, 6.00]
Outcomes (%)				
Death	108 (21.2)	120 (31.2)	46 (27.1)	1 (6.7)
Disability	2 (0.4)	1 (0.3)	1 (0.6)	0 (0.0)
Hospitalization	135 (26.5)	131 (34.0)	21 (12.4)	7 (46.7)
Life threatening	55 (10.8)	49 (12.7)	10 (5.9)	2 (13.3)
Other serious	191 (37.5)	67 (17.4)	77 (45.3)	5 (33.3)
Require intervention to prevent permanent	1 (0.2)	0 (0.0)	0 (0.0)	0 (0.0)
Missing	17 (3.3)	17 (4.4)	15 (8.8)	0 (0.0)

### Signals of system organ class

The system organ class distribution of AEs is shown in Supplementary Figure S1. Infections and infestations were most frequent for daunorubicin, doxorubicin and idarubicin, while cardiovascular disorders predominated for epirubicin. Table 2 presents the safety signal detection results across System Organ Classes (SOCs) using four pharmacovigilance methods (ROR, PRR, BCPNN, and MGPS). Strongest signals emerged for cardiac disorders (meeting ROR, PRR and EBGM05 criteria) and pregnancy/perinatal conditions (meeting all four criteria), with pregnancy-related AEs consistently significant across all methods. Partial positive signals were observed for infections and infestations (meeting only ROR and EBGM05 criteria) and reproductive/breast disorders (meeting ROR, PRR, and EBGM05 criteria but failing IC025 threshold). Other SOCs showed no significant safety signals due to either insufficient case numbers or failure to meet detection criteria.

**TABLE 2:** Signal strength of reports of anthracycline for treating acute myeloid leukemia at the system organ class level.

SOC	N	ROR (95%CI)	PRR (χ2)	EBGM (95%CI)	IC (95%CI)
Infections and infestations	737	1.58 (1.45–1.71)	1.46 (121.02)	1.45 (1.35–1.55)	0.53 (−1.13–2.2)
Blood and lymphatic system disorders	443	1.07 (0.97–1.19)	1.06 (1.92)	1.06 (0.98–1.16)	0.09 (−1.58–1.75)
General disorders and administration site conditions	353	0.63 (0.57–0.71)	0.67 (67.59)	0.67 (0.61–0.74)	−0.57 (−2.24–1.09)
Cardiac disorders	287	2.56 (2.26–2.9)	2.44 (238.64)	2.36 (2.13–2.62)	1.24 (−0.43–2.91)
Gastrointestinal disorders	257	0.99 (0.87–1.13)	0.99 (0.02)	0.99 (0.89–1.1)	−0.01 (−1.68–1.66)
Respiratory, thoracic and mediastinal disorders	254	1.41 (1.24–1.6)	1.38 (26.87)	1.37 (1.23–1.52)	0.45 (−1.22–2.12)
Investigations	241	0.58 (0.51–0.67)	0.61 (65.57)	0.62 (0.55–0.69)	−0.7 (−2.36–0.97)
Neoplasms benign, malignant and unspecified (inclcysts and polyps)	166	0.92 (0.79–1.08)	0.92 (1.1)	0.92 (0.81–1.05)	−0.11 (−1.78–1.55)
Nervous system disorders	161	1.13 (0.96–1.33)	1.13 (2.29)	1.12 (0.98–1.28)	0.17 (−1.5–1.83)
Injury, poisoning and procedural complications	135	0.56 (0.47–0.66)	0.58 (44.58)	0.58 (0.5–0.67)	−0.78 (−2.45–0.88)
Skin and subcutaneous tissue disorders	90	0.92 (0.75–1.14)	0.92 (0.57)	0.93 (0.78–1.1)	−0.11 (−1.78–1.56)
Vascular disorders	90	1.28 (1.03–1.58)	1.27 (5.15)	1.26 (1.06–1.51)	0.34 (−1.33–2)
Hepatobiliary disorders	86	1.24 (1–1.54)	1.24 (3.89)	1.23 (1.03–1.48)	0.3 (−1.37–1.97)
Metabolism and nutrition disorders	83	0.92 (0.74–1.15)	0.92 (0.53)	0.93 (0.77–1.11)	−0.11 (−1.78–1.55)
Renal and urinary disorders	58	0.87 (0.67–1.13)	0.87 (1.08)	0.87 (0.7–1.09)	−0.19 (−1.86–1.47)
Psychiatric disorders	36	0.94 (0.68–1.31)	0.94 (0.13)	0.94 (0.71–1.24)	−0.08 (−1.75–1.58)
Musculoskeletal and connective tissue disorders	33	0.6 (0.42–0.85)	0.6 (8.65)	0.61 (0.46–0.81)	−0.72 (−2.39–0.95)
Prenancy, puerperium and perinatalconditions	25	5.82 (3.83–8.83)	5.78 (87.78)	5.24 (3.69–7.43)	2.39 (0.71–4.07)
Eye disorders	21	1.02 (0.66–1.57)	1.02 (0.01)	1.02 (0.71–1.46)	0.03 (−1.64–1.7)
Immune system disorders	21	0.43 (0.28–0.66)	0.43 (15.95)	0.44 (0.3–0.62)	−1.2 (−2.87–0.47)
Surgical and medical procedures	14	0.17 (0.1–0.29)	0.17 (55.81)	0.18 (0.11–0.28)	−2.5 (−4.16–−0.83)
Reproductive system and breast disorders	12	2.19 (1.22–3.91)	2.18 (7.35)	2.13 (1.31–3.46)	1.09 (−0.59–2.77)
Congenital, familial and genetic disorders	6	1.03 (0.46–2.31)	1.03 (0)	1.03 (0.52–2.03)	0.04 (−1.64–1.72)
Ear and labyrinth disorders	3	0.69 (0.22–2.16)	0.69 (0.41)	0.7 (0.27–1.81)	−0.52 (−2.2–1.15)
Endocrine disorders	2	0.39 (0.1–1.57)	0.39 (1.89)	0.4 (0.12–1.27)	−1.34 (−3.01–0.34)

A comprehensive evaluation of safety signals for the four anthracyclines (Supplementary Table S3) demonstrated that epirubicin exhibited the strongest cardiotoxicity signal (ROR = 10.57, PRR = 8.01, EBGM05 = 57.83, IC025 = 1.29), while doxorubicin showed the most significant pregnancy-related adverse event signal (ROR = 9.97, PRR = 9.86, EBGM05 = 33.9, IC025 = 1.57), both consistently positive across all detection methods. Daunorubicin also showed significant associations for cardiotoxicity (ROR = 2.58, PRR = 2.45, EBGM05 = 86.98) and vascular disorders (ROR = 2.38, PRR = 2.32, EBGM05 = 39.6). Notably, idarubicin’s reproductive system adverse events (ROR = 4.0, PRR = 3.99, EBGM05 = 11.14) and epirubicin’s hepatobiliary toxicity (ROR = 4.99, PRR = 4.63, EBGM05 = 6.72) showed borderline significance with IC025 values of 0.28 and 0.5, respectively.

### Signals of preferred terms

**FIGURE 2:**
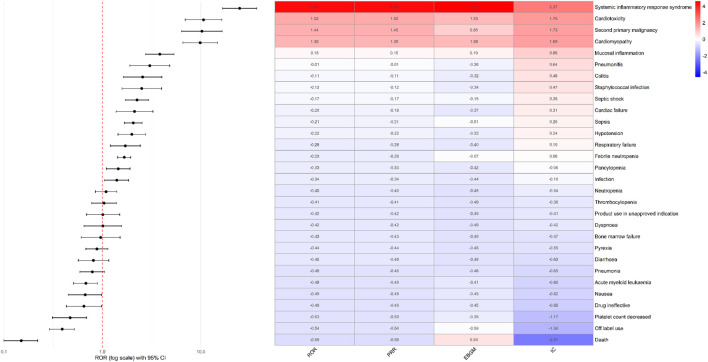
Forest plot and heatmap (top 30) of preferred terms induced by anthracycline for treating acute myeloid leukemia. BCPNN, Bayesian confidence propagation neural network; MGPS, multi-item gamma Poisson shrinker; PRR, proportional reported ratio; ROR, reporting odds ratio. Note: The number of reports differed substantially across anthracycline agents (e.g., epirubicin n = 15 vs. idarubicin n = 509). Estimates based on small case numbers may be unstable and should be interpreted with caution.

Figure 2 displays the adverse event signals for specific PTs across all anthracyclines. The strongest positive signals were observed for systemic inflammatory response syndrome (ROR = 24.79, PRR = 24.54, EBGM05 = 386.91, IC025 = 2.33), cardiomyopathy (ROR = 9.83, PRR = 9.76, EBGM05 = 133.98, IC025 = 1.35), and cardiotoxicity (ROR = 10.63, PRR = 10.57, EBGM05 = 127.59, IC025 = 1.44), all consistently positive across methods. Mucosal inflammation (ROR = 3.83, PRR = 3.8, EBGM05 = 54.91, IC025 = 0.17) and septic shock (ROR = 2.25, PRR = 2.23, EBGM05 = 28.4, IC025 = −0.55) also showed significant associations, though the latter’s IC025 was slightly below threshold. Interestingly, febrile neutropenia (ROR = 1.67, PRR = 1.63, EBGM05 = 38.68) and pancytopenia (ROR = 1.45, PRR = 1.45, EBGM05 = 5.69) met some criteria but had negative IC025 values (−0.98 and −1.15, respectively). Other adverse events like neutropenia (ROR = 1.09) and thrombocytopenia (ROR = 1.04) were not considered strong positive signals due to incomplete criteria fulfillment.

**FIGURE 3:**
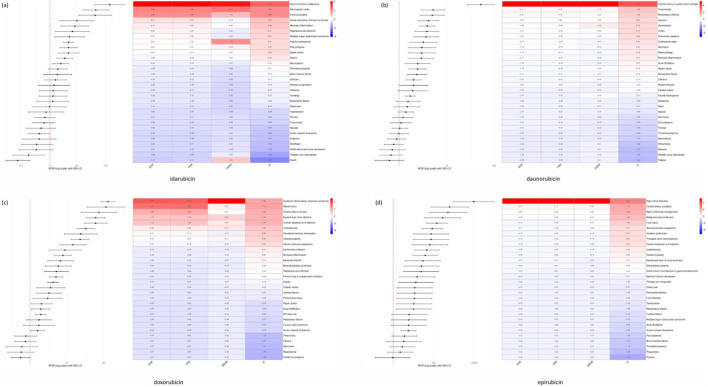
Forest plot and heatmap (top 30) of preferred terms induced by idarubicin **(a)**, daunorubicin **(b)**, doxorubicin **(c)**, and epirubicin **(d)** for treating acute myeloid leukemia. BCPNN, Bayesian confidence propagation neural network; MGPS, multi-item gamma Poisson shrinker; PRR, proportional reported ratio; ROR, reporting odds ratio. Note: The number of reports differed substantially across anthracycline agents (e.g., epirubicin n = 15 vs. idarubicin n = 509). Estimates based on small case numbers may be unstable and should be interpreted with caution.

Figure 3 presents drug-specific safety signals for individual PTs. Doxorubicin-associated systemic inflammatory response syndrome showed exceptionally strong signals (ROR = 132.06, PRR = 125.07, EBGM05 = 1998.16, IC025 = 4.71), as did epirubicin-associated right atrial dilation (ROR = 9449.66). Cardiotoxicity was particularly prominent for doxorubicin (ROR = 32.86) and epirubicin (ROR = 45.76), while daunorubicin showed significant central nervous system hemorrhage (ROR = 52.42) and idarubicin demonstrated neutropenic colitis (ROR = 6.68).

### Onset time of events

**FIGURE 4:**
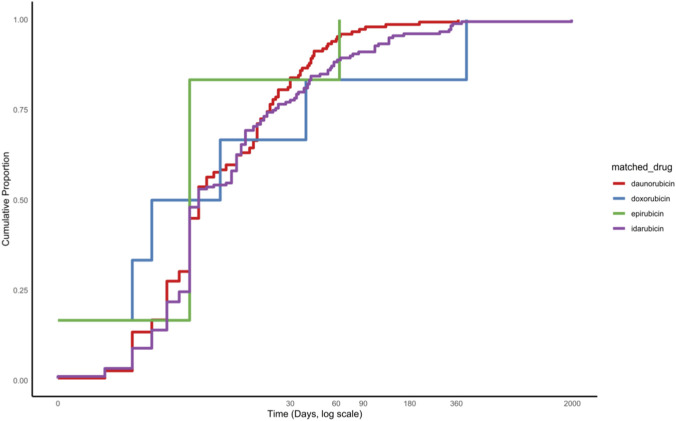
The cumulative risk curve of time to induce adverse events for anthracycline. Note: 1) The x-axis shows time (in days) on a base-10 logarithmic scale to highlight early events. 2) The number of reports differed substantially across anthracycline agents (e.g., epirubicin n = 15 vs. idarubicin n = 509). Estimates based on small case numbers may be unstable and should be interpreted with caution.

**FIGURE 5:**
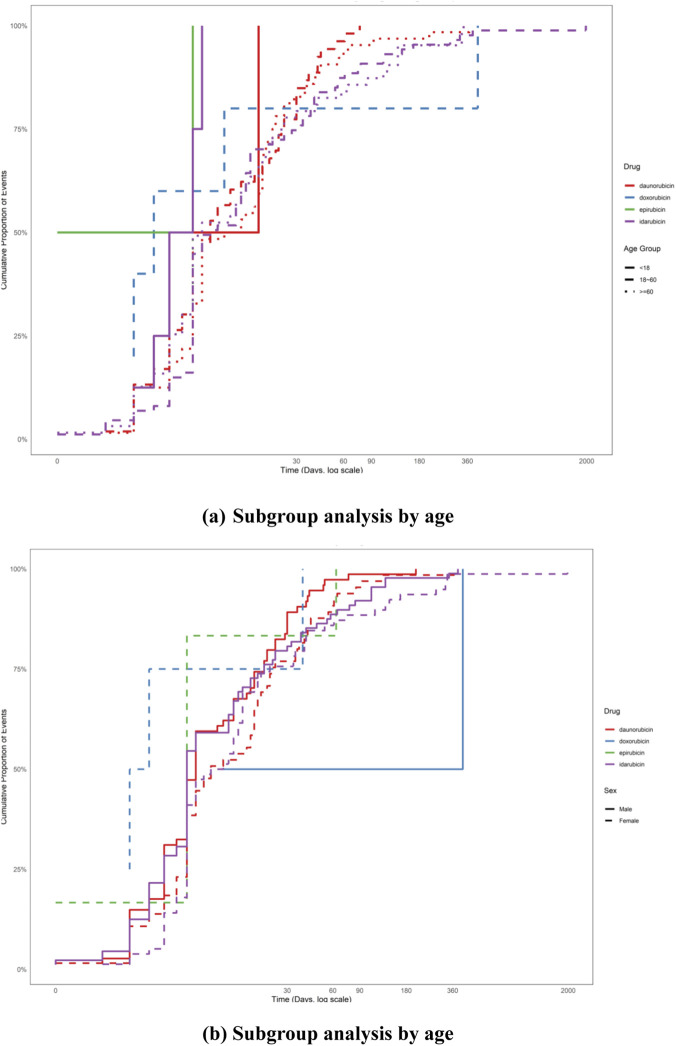
The cumulative risk curve of time to induce adverse events for anthracycline by age **(a)** and sex **(b)**. Note: 1) The x-axis shows time (in days) on a base-10 logarithmic scale to highlight early events. 2) The number of reports differed substantially across anthracycline agents (e.g., epirubicin n = 15 vs. idarubicin n = 509). Estimates based on small case numbers may be unstable and should be interpreted with caution.

The cumulative hazard curve (Figure 4) demonstrated that the majority of adverse events occurred within the first 180 days following treatment initiation, with no statistically significant differences observed between the different drug groups (χ^2^ = 1.39, *P* = 0.71). Figure 5a showed the further stratification by age group (<18, 18–60, and ≥60 years) of time-to-onset of adverse events. Specifically, idarubicin exhibited a significantly longer time-to-onset in patients aged 60 years or older compared to those under 18 years (*P* < 0.05). In contrast, no significant age-related differences were observed for the other drugs analyzed. Gender-based analysis (Figure 5b) uncovered differential drug effects between males and females. Doxorubicin was associated with a longer time-to-onset of adverse events in male patients compared to females (*P* = 0.04), whereas idarubicin showed the opposite trend, with a shorter time-to-onset in males (*P* = 0.049). The remaining drugs did not exhibit significant gender-related differences.

## Discussion

This study examined the AE profile of anthracycline drugs in AML treatment and analyze the incidence and distribution patterns of adverse reactions across different anthracycline agents and patient populations using FAERS database. Signal detection identified pregnancy-related, cardiac, systemic inflammation, and cardiotoxicity as key safety signals with drug-specific risks. Time-to-onset analysis indicated that doxorubicin may be a better choice for males, and idarubicin for females.

Our findings in AEs profiles of anthracyclines generally align with established literature (Narezkina et al., 2021; Wood, 2004). Anthracyclines predominantly induce systemic inflammatory responses and cardiotoxicity, followed by infection-related complications. The strong association with cardiotoxicity is well-documented, particularly for doxorubicin, which disrupts mitochondrial function and promotes oxidative stress (Narezkina et al., 2021; Kobza, 2021). Recent reviews and cohort studies provide additional context: a recent JACC CardioOncology review notes that most anthracycline cardiotoxicity occurs within the first year (early-onset) (Camilli et al., 2024), and 2026 AML-specific data highlight differences in heart failure incidence across anthracycline agents (Geels et al., 2026). It should be noted that the observed signal strength in FAERS reflects reporting frequency rather than absolute risk, and may be influenced by factors such as reporting bias, concomitant therapies, or patient characteristics. Similarly, systemic inflammatory reactions, such as cytokine release syndrome, have been reported in patients receiving high-dose regimens (Grant et al., 2020). These consistent signals underscore the need for vigilant monitoring in clinical practice, especially for patients with preexisting cardiovascular risk factors.

This study reveals the specific toxicity patterns among anthracyclines, providing targeted options for drug therapy in AML. Among them, doxorubicin’s significant systemic inflammation and cardiotoxicity pose greater harm to patients and are currently the most common complications in clinical medication (Shi et al., 2023; Song et al., 2024). Daunorubicin’s bleeding tendency may stem from its stronger bone marrow suppression (Zhang et al., 2022). Existing studies show inconsistent results. Seipelt et al. found less myelosuppression than idarubicin in 27 patients (Cottin et al., 1998), while Zhang et al. reported similar bleeding rates in 58 patients (Zhang et al., 2022). These discrepancies may be due to the relatively small sample sizes in previous studies, warranting further investigation with larger cohorts. Idarubicin’s infection risk is consistent with its strong inhibitory effect on myeloid cells (DiNardo et al., 2021). Although epirubicin’s extreme vascular toxicity is rare, it is consistent with case reports of coronary artery spasm (Liang et al., 2021). Such adverse reactions may be related to drug-induced vascular endothelial dysfunction (Zhang et al., 2002), and the risk is significantly higher in patients with a history of coronary heart disease or smoking (Fogarassy et al., 2019). For patients with risk factors for vascular diseases such as hypertension and hyperlipidemia, switching to other anthracyclines or using continuous intravenous infusion to reduce peak concentration-related toxicity may be considered (Buchalska et al., 2025). It should be noted that sample sizes varied considerably across anthracycline agents, with epirubicin represented by only 15 cases, while idarubicin included 509 cases. For epirubicin, the high female proportion and small case number may reflect reporting from non-AML indications, and indication misclassification cannot be excluded. This further limits the precision of effect estimates and increases uncertainty around observed signals.

Our time-to-event analyses revealed clinically meaningful age and sex disparities in anthracycline toxicity profiles. However, some subgroups contained few cases, which may limit the precision of these stratified analyses. Idarubicin demonstrated a significantly longer time-to-onset of adverse events in patients over 60 years compared to those under 18 years, which may suggest age-related variations in toxicity patterns rather than definitive therapeutic suitability. This aligns with pharmacokinetic studies demonstrating age-related reductions in hepatic metabolism (Cadeddu Dessalvi et al., 2019; Tedjaseputra et al., 2025) and real-world data showing older patients experience more frequent grade 3/4 toxicities (33.9% vs. 10.7%) yet most complete treatment (Monteiro et al., 2021). However, the underlying mechanisms cannot be determined from spontaneous reporting data. The delayed toxicity may reflect decreased renal clearance prolonging drug retention (Polonsky and DeCara, 2019). Notably, idarubicin showed a comparatively delayed TTO signal in elderly patients, given its potentially lower cardiotoxicity risk versus other anthracyclines (Megías-Vericat et al., 2021). The mechanisms underlying this age effects may be due to declining organ function (Polonsky and DeCara, 2019), but recent work suggests body composition metrics (e.g., lean muscle mass) independently predict toxicity risk (Qiao et al., 2024). In addition, sex differences emerged equally prominently. Females demonstrated greater cardiotoxicity susceptibility, consistent with estrogen’s known cardioprotective effects (Cadeddu Dessalvi et al., 2019). This pattern was most evident with idarubicin, which showed shorter time-to-onset in males (Meng et al., 2025). Conversely, doxorubicin exhibited prolonged induction time in males, likely reflecting higher CYP3A4 activity enhancing metabolism (Puty et al., 2019). While hormonal and metabolic factors have been proposed in previous studies (Cadeddu Dessalvi et al., 2019; Puty et al., 2019), these explanations cannot be confirmed within spontaneous reporting systems. In addition, sex-stratified findings may be influenced by non-random missingness and reporting bias, rather than true incidence. For example, sex information was missing for 35.3% of doxorubicin cases and 13.4% of idarubicin cases. Therefore, these findings suggest potential sex-related variations in AE patterns, but should be interpreted as exploratory and hypothesis-generating rather than guiding definitive clinical recommendations.

This study has several important limitations. First, while the FAERS database provides extensive coverage, its voluntary reporting system leads to inherent issues including underreporting, delayed submissions, and missing critical patient information (e.g., age, gender), potentially introducing bias. Observed event counts, such as the higher number of doxorubicin reports, may reflect reporting frequency or drug usage rather than true per-patient risk, potentially biasing comparative safety interpretations. Second, disproportionality analysis can only assess drug–adverse event reporting associations rather than establish causality and does not allow adjustment for comorbidities, concomitant medications, disease severity, or treatment line. Differential prescribing patterns across patient subgroups may introduce indication bias, such that observed differences in reporting patterns may reflect underlying patient characteristics rather than intrinsic drug properties. Third, our analysis was restricted to data predominantly from Western populations, which may limit generalizability to other ethnic groups. Differences in pharmacogenetics, treatment practices, and adverse event reporting over the 20-year study period could introduce geographic and temporal variability. Further pharmacovigilance studies in non-Western cohorts are needed to assess the applicability of our findings. Last, FAERS lacks detailed clinical information, including comorbidities, disease severity, treatment line, concomitant medications, and standardized data on cumulative drug exposure, which precludes adjustment for potential confounders and assessment of dose–response relationships. As anthracyclines are typically administered within multi-agent AML regimens, reported adverse events may partly reflect co-administered therapies or underlying patient characteristics rather than the anthracycline itself. Certain anthracyclines may be preferentially prescribed to sicker patients or those with specific comorbidities, introducing potential indication bias that could influence observed AE patterns. Therefore, these signals should be interpreted cautiously and regarded as hypothesis-generating rather than causal. Despite these constraints, disproportionality analysis remains valuable for initial signal detection. Our findings should be interpreted as preliminary, warranting confirmation through prospective studies or targeted cohort analyses.

## Conclusion

This study indicates that the safety profile of anthracycline for acute myeloid leukemia treatment using the FAERS database. Anthracyclines demonstrate distinct toxicity profiles: epirubicin and doxorubicin for cardiac/pregnancy risks; doxorubicin for systemic/cardiac effects; daunorubicin for hemorrhagic complications; and idarubicin for myelosuppression/infections, with systemic inflammation representing a major class-wide safety concern. Observed time-to-onset differences indicated that idarubicin tended to have delayed onset in elderly patients, while doxorubicin showed prolonged toxicity onset in males and accelerated onset of idarubicin in males versus females. These patterns suggest potential age- and sex-related variations in AE profiles, but should be interpreted as preliminary signals and hypothesis-generating rather than definitive clinical recommendations. Overall, these findings provide real-world insights into anthracycline safety in AML and warrant further investigation in prospective studies or targeted cohort analyses.

## Data Availability

The datasets presented in this study can be found in online repositories. The names of the repository/repositories and accession number(s) can be found below: https://www.fda.gov/drugs/drug-approvals-and-databases/fda-adverse-event-reporting-system-faers-database.

## References

[B1] Bhagat A. Kleinerman E. S. (2020). Anthracycline-induced cardiotoxicity: causes, mechanisms, and prevention. Adv. Exp. Med. Biol. 1257, 181–192. 10.1007/978-3-030-43032-0_15 32483740

[B2] Buchalska B. Kamińska K. Kowara M. Sobiborowicz-Sadowska A. Cudnoch-Jędrzejewska A. (2025). Doxorubicin or epirubicin *versus* liposomal doxorubicin therapy-differences in cardiotoxicity. Cardiovasc Toxicol. 25 (2), 248–268. 10.1007/s12012-024-09952-4 39810066

[B3] Cadeddu Dessalvi C. Pepe A. Penna C. Gimelli A. Madonna R. Mele D. (2019). Sex differences in anthracycline-induced cardiotoxicity: the benefits of estrogens. Heart Fail Rev. 24 (6), 915–925. 10.1007/s10741-019-09820-2 31256318

[B4] Camilli M. Cipolla C. M. Dent S. Minotti G. Cardinale D. M. (2024). Anthracycline cardiotoxicity in adult cancer patients: JACC: cardiooncology state-of-the-art review. JACC CardioOncol 6 (5), 655–677. 10.1016/j.jaccao.2024.07.016 39479333 PMC11520218

[B5] Cottin Y. Touzery C. Dalloz F. Coudert B. Toubeau M. Riedinger A. (1998). Comparison of epirubicin and doxorubicin cardiotoxicity induced by low doses: evolution of the diastolic and systolic parameters studied by radionuclide angiography. Clin. Cardiol. 21 (9), 665–670. 10.1002/clc.4960210911 9755384 PMC6655270

[B6] Dai Z. Wang G. Zhang J. Zhao Q. Jiang L. (2024). Adverse events associated with eteplirsen: a disproportionality analysis using the 2016-2023 FAERS data. Heliyon 10 (13), e33417. 10.1016/j.heliyon.2024.e33417 39027557 PMC11255655

[B7] Dantchev D. Balercia G. Bourut C. Anjo A. Maral R. Mathé G. (1984). Comparative microscopic study of cardiotoxicity and skin toxicity of anthracycline analogs. Biomed. Pharmacother. 38 (7), 322–328. 6240996

[B8] Dazzi H. Kaufmann K. Follath F. (2001). Anthracycline-induced acute cardiotoxicity in adults treated for leukaemia. Analysis of the clinico-pathological aspects of documented acute anthracycline-induced cardiotoxicity in patients treated for acute leukaemia at the university hospital of Zürich, Switzerland, between 1990 and 1996. Ann. Oncol. 12 (7), 963–966. 10.1023/a:1011196910325 11521803

[B9] DiNardo C. D. Lachowiez C. A. Takahashi K. Loghavi S. Xiao L. Kadia T. (2021). Venetoclax combined with FLAG-IDA induction and consolidation in newly diagnosed and relapsed or refractory acute myeloid leukemia. J. Clin. Oncol. 39 (25), 2768–2778. 10.1200/JCO.20.03736 34043428 PMC8407653

[B10] Fogarassy G. Vathy-Fogarassy Á. Kenessey I. Kásler M. Forster T. (2019). Risk prediction model for long-term heart failure incidence after epirubicin chemotherapy for breast cancer - a real-world data-based, nationwide classification analysis. Int. J. Cardiol. 285, 47–52. 10.1016/j.ijcard.2019.03.013 30905520

[B11] Geels J. van Rhenen A. Gradowska P. Asselbergs F. W. Linschoten M. (2026). Heart failure in patients with acute myeloid leukemia (AML) treated with anthracycline agents during remission induction therapy: a systematic review and meta-analysis. Leukemia 40 (1), 120–129. 10.1038/s41375-025-02753-w 41073560

[B12] Grant M. K. O. Abdelgawad I. Y. Lewis C. A. Zordoky B. N. (2020). Sexual dimorphism in doxorubicin-induced systemic inflammation: implications for hepatic cytochrome P450 regulation. Int. J. Mol. Sci. 21 (4). 10.3390/ijms21041279 32074957 PMC7072970

[B13] Hauben M. Zou C. Bright S. Hung E. (2021). More extreme duplication in the U.S. FDA FAERS database and a suggested check point for disproportionality analysis. Pharmacoepidemiol Drug Saf. 30 (8), 1140–1141. 10.1002/pds.5265 33960586

[B14] Hefti E. Blanco J. G. (2016). Anthracycline-related cardiotoxicity in patients with acute myeloid leukemia and Down syndrome: a literature review. Cardiovasc Toxicol. 16 (1), 5–13. 10.1007/s12012-015-9307-1 25616318 PMC4514565

[B15] Kobza C. (2021). Cardiac toxicity: using angiotensin-converting enzyme inhibitors to prevent anthracycline-induced left ventricular dysfunction and cardiomyopathy. Clin. J. Oncol. Nurs. 25 (3), 259–266. 10.1188/21.CJON.259-266 34019025

[B16] Liang H. Z. Zhao H. Gao J. Cao C. F. Wang W. M. (2021). Epirubicin-induced kounis syndrome. BMC Cardiovasc Disord. 21 (1), 133. 10.1186/s12872-021-01936-4 33711934 PMC7953621

[B17] Lin G. Chen R. Wen C. Li Z. Yan X. Li L. (2025). Analyzing real-world adverse events of spironolactone with the FAERS database. PLoS One 20 (9), e0330659. 10.1371/journal.pone.0330659 40961031 PMC12443299

[B18] Lindquist M. Ståhl M. Bate A. Edwards I. R. Meyboom R. H. (2000). A retrospective evaluation of a data mining approach to aid finding new adverse drug reaction signals in the WHO international database. Drug Saf. 23 (6), 533–542. 10.2165/00002018-200023060-00004 11144660

[B19] Mattioli R. Ilari A. Colotti B. Mosca L. Fazi F. Colotti G. (2023). Doxorubicin and other anthracyclines in cancers: activity, chemoresistance and its overcoming. Mol. Asp. Med. 93, 101205. 10.1016/j.mam.2023.101205 37515939

[B20] McGowan J. V. Chung R. Maulik A. Piotrowska I. Walker J. M. Yellon D. M. (2017). Anthracycline chemotherapy and cardiotoxicity. Cardiovasc Drugs Ther. 31 (1), 63–75. 10.1007/s10557-016-6711-0 28185035 PMC5346598

[B21] Megías-Vericat J. E. Martínez-Cuadrón D. Herrero M. J. Rodríguez-Veiga R. Solana-Altabella A. Boluda B. (2021). Impact of combinations of single-nucleotide polymorphisms of anthracycline transporter genes upon the efficacy and toxicity of induction chemotherapy in acute myeloid leukemia. Leuk. Lymphoma 62 (3), 659–668. 10.1080/10428194.2020.1839650 33135528

[B22] Meng F. Xiang M. Xie H. Liu Y. Qi Y. Zeng D. (2025). Retrospective study on the clinical outcomes and characteristics of acute myeloid leukemia: different outcomes in the same risk group. PeerJ 13, e20436. 10.7717/peerj.20436 41368499 PMC12684409

[B23] Monteiro A. R. Garcia A. R. Póvoa S. Soares R. F. Macedo F. Pereira T. C. (2021). Acute toxicity and tolerability of anthracycline-based chemotherapy regimens in older *versus* younger patients with breast cancer: real-world data. Support Care Cancer 29 (5), 2347–2353. 10.1007/s00520-020-05766-6 32918130

[B24] Narezkina A. Narayan H. K. Zemljic-Harpf A. E. (2021). Molecular mechanisms of anthracycline cardiovascular toxicity. Clin. Sci. (Lond) 135 (10), 1311–1332. 10.1042/CS20200301 34047339 PMC10866014

[B25] Neuendorff N. R. Loh K. P. Mims A. S. Christofyllakis K. Soo W. K. Bölükbasi B. (2020). Anthracycline-related cardiotoxicity in older patients with acute myeloid leukemia: a young SIOG review paper. Blood Adv. 4 (4), 762–775. 10.1182/bloodadvances.2019000955 32097461 PMC7042993

[B26] Polonsky T. S. DeCara J. M. (2019). Risk factors for chemotherapy-related cardiac toxicity. Curr. Opin. Cardiol. 34 (3), 283–288. 10.1097/HCO.0000000000000619 30870259

[B27] Puty T. C. Sarraf J. S. Do Carmo Almeida T. C. Filho V. C. B. de Carvalho L. E. W. Fonseca F. L. A. (2019). Evaluation of the impact of single-nucleotide polymorphisms on treatment response, survival and toxicity with cytarabine and anthracyclines in patients with acute myeloid leukaemia: a systematic review protocol. Syst. Rev. 8 (1), 109. 10.1186/s13643-019-1011-y 31053175 PMC6499963

[B28] Qiao X. van der Zanden S. Y. Li X. Tan M. Zhang Y. Song J. Y. (2024). Diversifying the anthracycline class of anti-cancer drugs identifies aclarubicin for superior survival of acute myeloid leukemia patients. Mol. Cancer 23 (1), 120. 10.1186/s12943-024-02034-7 38831402 PMC11149191

[B29] Sakaeda T. Tamon A. Kadoyama K. Okuno Y. (2013). Data mining of the public version of the FDA adverse event reporting system. Int. J. Med. Sci. 10 (7), 796–803. 10.7150/ijms.6048 23794943 PMC3689877

[B30] Savani M. Woerner K. Bu L. Birkenbach M. Skubitz K. M. (2021). Pegylated liposomal doxorubicin-induced renal toxicity in retroperitoneal liposarcoma: a case report and literature review. Cancer Chemother. Pharmacol. 87 (2), 289–294. 10.1007/s00280-020-04203-z 33388949

[B31] Schlenk R. F. (2023). Acute myeloid leukemia: introduction to a series highlighting progress and ongoing challenges. Haematologica 108 (2), 306–307. 10.3324/haematol.2022.280803 36722401 PMC9890027

[B32] Sheibani M. Azizi Y. Shayan M. Nezamoleslami S. Eslami F. Farjoo M. H. (2022). Doxorubicin-induced cardiotoxicity: an overview on pre-clinical therapeutic approaches. Cardiovasc Toxicol. 22 (4), 292–310. 10.1007/s12012-022-09721-1 35061218

[B33] Shi S. Chen Y. Luo Z. Nie G. Dai Y. (2023). Role of oxidative stress and inflammation-related signaling pathways in doxorubicin-induced cardiomyopathy. Cell Commun. Signal 21 (1), 61. 10.1186/s12964-023-01077-5 36918950 PMC10012797

[B34] Song L. Qiu Q. Ju F. Zheng C. (2024). Mechanisms of doxorubicin-induced cardiac inflammation and fibrosis; therapeutic targets and approaches. Arch. Biochem. Biophys. 761, 110140. 10.1016/j.abb.2024.110140 39243924

[B35] Tedjaseputra A. Tey A. Nalpantidis A. Grigoriadis G. Fleming S. Vilcassim S. (2025). Ratifying the efficacy and safety of intensive induction chemotherapy for acute myeloid leukaemia by the australasian leukaemia and lymphoma group consensus approach. Intern Med. J. 55 (5), 749–759. 10.1111/imj.70010 40052282 PMC12077584

[B36] Tuzovic M. Wu P. T. Kianmahd S. Nguyen K. L. (2018). Natural history of myocardial deformation in children, adolescents, and young adults exposed to anthracyclines: systematic review and meta-analysis. Echocardiography 35 (7), 922–934. 10.1111/echo.13871 29603386 PMC6544758

[B37] Venugopal S. Sekeres M. A. (2024). Contemporary management of acute myeloid leukemia: a review. JAMA Oncol. 10 (10), 1417–1425. 10.1001/jamaoncol.2024.2662 39115831

[B38] Wood L. S. (2004). Liposomal anthracycline administration and toxicity management: a nursing perspective. Semin. Oncol. 31 (6), 182–190. 10.1053/j.seminoncol.2004.08.007 15717743

[B39] Zhang X. S. Zhu X. F. Gao J. S. Ye Y. L. Feng Q. S. Liu Z. C. (2002). Variable sensitivity of endothelial cells to epirubicin in xenografts of human nasopharyngeal carcinoma CNE-2 cells. Cancer Biol. Ther. 1 (3), 263–265. 10.4161/cbt.78 12432274

[B40] Zhang Q. Zhang C. H. Wang Z. D. Wang D. (2022). Efficacy and safety of induction chemotherapy with daunorubicin or idarubicin in the treatment of an adult with acute lymphoblastic leukemia. Tumori 108 (2), 182–188. 10.1177/03008916211032724 34296648

