# Supplementary material for: Post-marketing safety evaluation of anthracycline for acute myeloid leukemia treatment: a real-world pharmacovigilance analysis
Source: Front Pharmacol. 2026 Jun 10;17:1759201. doi: 10.3389/fphar.2026.1759201 (PMC13290724; doi:10.3389/fphar.2026.1759201)
Supplement: Supplementary file 1 [file Supplementaryfile1.docx]

**
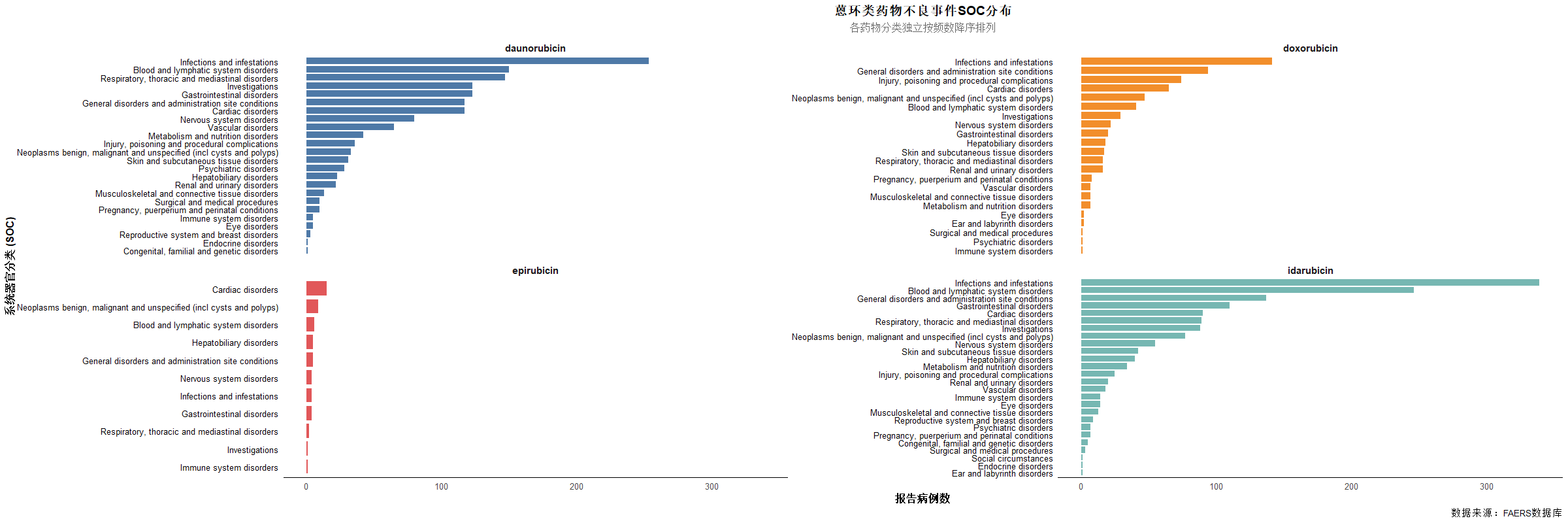
**

**Supplementary Figure 1. The distribution of** **adverse events in the** **system organ class induced by** **anthracycline for treating acute myeloid leukemia.**

**Supplementary Table 1 Four-grid table of disproportionality analysis method.**

| **Item** | **Target adverse events** | **All other adverse events** | **Total** |
| --- | --- | --- | --- |
| Target drugs | a | b | a + b |
| All other drugs | c | d | c + d |
| Total | a + c | b + d | a + b + c + d |

**Supplementary Table 2 Principle of disproportionality measure and standard of signal detection.**

| **Methods** | **Calculation formula** | **Criteria for positive signals [1, 2]** |
| --- | --- | --- |
| ROR | ROR = ad/bc | Lower limit of 95% CI > 1, N ≥ 3 |
|  | 95%CI = e^ln(ROR)±1.96(1/a+1/b+1/c+1/d)^0.5^ |  |
| PRR | PRR = [a(c+d)]/[c(a+b)] | PRR ≥ 2, *χ^2^*≥ 4, N ≥ 3 |
|  | *χ^2^* =[(ad-bc)^2](a+b+c+d)/[(a+b)(c+d)(a+c)(b+d)] |  |
| BCPNN | IC = log_2_a(a+b+c+d)/[(a+c)(a+b)] | IC025 > 0 |
|  | 95%*CI* = E(IC) ± 2[V(IC)]^0.5 |  |
| MGPS | EBGM = a(a+b+c+d)/[(a+c)(a+b)] | EBGM05 > 2 |
|  | 95%*CI* = e^ln(EBGM)±1.96(1/a+1/b+1/c+1/d)^0.5^ |  |

95%CI, 95% confidence interval; N, the number of reports; χ2, chi-squared; IC, information component; IC025, the lower limit of 95% CI of the IC; E(IC), the IC expectations; V(IC), the variance of IC; EBGM, empirical Bayesian geometric mean; EBGM05, the lower limit of 95% CI of EBGM.

**Supplementary Table 3 Signal strength of reports of** **idarubicin, daunorubicin, doxorubicin, and epirubicin for treating acute myeloid leukemia at the system organ class level**

| Drug name | SOC | N | ROR (95%CI) | PRR (χ2) | EBGM (95%CI) | IC (95%CI) |
| --- | --- | --- | --- | --- | --- | --- |
| idarubicin | Infections and infestations | 339 | 1.81 (1.61 - 2.05) | 1.63 (94.19) | 1.62(1.46-1.79) | 0.7 (-0.97 - 2.36) |
|  | Blood and lymphatic system disorders | 246 | 1.53 (1.33 - 1.75) | 1.44 (37.13) | 1.44(1.28-1.61) | 0.52 (-1.15 - 2.19) |
|  | General disorders and administration site conditions | 137 | 0.6 (0.5 - 0.71) | 0.63 (34.08) | 0.63(0.55-0.74) | -0.66 (-2.32 - 1.01) |
|  | Gastrointestinal disorders | 110 | 1.04 (0.85 - 1.26) | 1.03 (0.13) | 1.03(0.88-1.22) | 0.05 (-1.62 - 1.72) |
|  | Cardiac disorders | 90 | 1.87 (1.51 - 2.32) | 1.82 (33.57) | 1.8(1.51-2.16) | 0.85 (-0.82 - 2.52) |
|  | Respiratory, thoracic and mediastinal disorders | 89 | 1.18 (0.95 - 1.46) | 1.17 (2.2) | 1.16(0.97-1.39) | 0.22 (-1.45 - 1.89) |
|  | Investigations | 88 | 0.52 (0.42 - 0.64) | 0.55 (37.06) | 0.55(0.46-0.66) | -0.87 (-2.53 - 0.8) |
|  | Neoplasms benign, malignant and unspecified (incl cysts and polyps) | 77 | 1.05 (0.83 - 1.32) | 1.04 (0.15) | 1.04(0.86-1.27) | 0.06 (-1.61 - 1.73) |
|  | Nervous system disorders | 55 | 0.93 (0.71 - 1.22) | 0.93 (0.28) | 0.93(0.74-1.17) | -0.1 (-1.77 - 1.57) |
|  | Skin and subcutaneous tissue disorders | 42 | 1.05 (0.77 - 1.43) | 1.05 (0.11) | 1.05(0.81-1.36) | 0.07 (-1.6 - 1.74) |
|  | Hepatobiliary disorders | 40 | 1.41 (1.03 - 1.93) | 1.4 (4.56) | 1.39(1.07-1.81) | 0.48 (-1.19 - 2.15) |
|  | Metabolism and nutrition disorders | 34 | 0.92 (0.65 - 1.29) | 0.92 (0.23) | 0.92(0.69-1.23) | -0.12 (-1.79 - 1.55) |
|  | Injury, poisoning and procedural complications | 25 | 0.25 (0.17 - 0.37) | 0.26 (56.15) | 0.26(0.19-0.36) | -1.93 (-3.6 - -0.27) |
|  | Renal and urinary disorders | 20 | 0.73 (0.47 - 1.13) | 0.73 (1.99) | 0.73(0.51-1.06) | -0.45 (-2.11 - 1.22) |
|  | Vascular disorders | 18 | 0.61 (0.38 - 0.97) | 0.61 (4.48) | 0.61(0.42-0.91) | -0.7 (-2.37 - 0.97) |
|  | Eye disorders | 14 | 1.67 (0.98 - 2.83) | 1.66 (3.65) | 1.65(1.06-2.57) | 0.72 (-0.95 - 2.39) |
|  | Immune system disorders | 14 | 0.7 (0.41 - 1.19) | 0.7 (1.76) | 0.71(0.45-1.1) | -0.5 (-2.17 - 1.17) |
|  | Musculoskeletal and connective tissue disorders | 13 | 0.58 (0.33 - 1) | 0.58 (3.98) | 0.58(0.37-0.92) | -0.78 (-2.45 - 0.89) |
|  | Reproductive system and breast disorders | 9 | 4 (2.06 - 7.8) | 3.99 (19.46) | 3.88(2.22-6.78) | 1.96 (0.28 - 3.63) |
|  | Pregnancy, puerperium and perinatal conditions | 7 | 3.67 (1.72 - 7.8) | 3.65 (13.08) | 3.57(1.9-6.71) | 1.84 (0.16 - 3.51) |
|  | Psychiatric disorders | 7 | 0.44 (0.21 - 0.93) | 0.44 (4.91) | 0.45(0.24-0.83) | -1.16 (-2.83 - 0.5) |
|  | Congenital, familial and genetic disorders | 5 | 2.11 (0.87 - 5.12) | 2.11 (2.85) | 2.08(0.99-4.38) | 1.06 (-0.62 - 2.74) |
|  | Surgical and medical procedures | 3 | 0.09 (0.03 - 0.28) | 0.09 (27.56) | 0.09(0.04-0.24) | -3.43 (-5.1 - -1.77) |
|  | Endocrine disorders | 1 | 0.48 (0.07 - 3.42) | 0.48 (0.56) | 0.48(0.09-2.49) | -1.05 (-2.73 - 0.62) |
|  | Ear and labyrinth disorders | 1 | 0.56 (0.08 - 4.01) | 0.56 (0.34) | 0.56(0.11-2.92) | -0.83 (-2.51 - 0.85) |
|  | Social circumstances | 1 | 0.51 (0.07 - 3.64) | 0.51 (0.47) | 0.51(0.1-2.65) | -0.97 (-2.64 - 0.71) |
| epirubicin | Cardiac disorders | 15 | 10.57 (5.85 - 19.11) | 8.01 (94.91) | 7.99(4.87-13.11) | 3 (1.29 - 4.71) |
|  | Neoplasms benign, malignant and unspecified (incl cysts and polyps) | 9 | 3.67 (1.8 - 7.5) | 3.24 (14.69) | 3.24(1.78-5.89) | 1.7 (-0.01 - 3.41) |
|  | Blood and lymphatic system disorders | 6 | 0.92 (0.4 - 2.15) | 0.93 (0.03) | 0.93(0.46-1.89) | -0.1 (-1.81 - 1.61) |
|  | General disorders and administration site conditions | 5 | 0.58 (0.23 - 1.45) | 0.62 (1.4) | 0.62(0.29-1.33) | -0.7 (-2.41 - 1.01) |
|  | Hepatobiliary disorders | 5 | 4.99 (1.99 - 12.51) | 4.63 (14.51) | 4.63(2.15-9.99) | 2.21 (0.5 - 3.92) |
|  | Infections and infestations | 4 | 0.47 (0.17 - 1.3) | 0.51 (2.22) | 0.51(0.22-1.19) | -0.98 (-2.69 - 0.73) |
|  | Nervous system disorders | 4 | 1.87 (0.67 - 5.16) | 1.8 (1.49) | 1.8(0.77-4.22) | 0.85 (-0.86 - 2.56) |
|  | Gastrointestinal disorders | 4 | 1 (0.36 - 2.76) | 1 (0) | 1(0.43-2.34) | 0 (-1.71 - 1.71) |
|  | Respiratory, thoracic and mediastinal disorders | 2 | 0.68 (0.17 - 2.81) | 0.7 (0.28) | 0.7(0.21-2.27) | -0.52 (-2.23 - 1.19) |
|  | Investigations | 1 | 0.15 (0.02 - 1.09) | 0.17 (4.71) | 0.17(0.03-0.87) | -2.59 (-4.3 - -0.88) |
|  | Immune system disorders | 1 | 1.35 (0.19 - 9.74) | 1.34 (0.09) | 1.34(0.26-7.02) | 0.42 (-1.29 - 2.13) |
| doxorubicin | Infections and infestations | 141 | 1.74 (1.44 - 2.1) | 1.58 (34.48) | 1.57(1.35-1.84) | 0.65 (-1.02 - 2.32) |
|  | General disorders and administration site conditions | 94 | 1.02 (0.82 - 1.27) | 1.02 (0.03) | 1.02(0.85-1.22) | 0.02 (-1.64 - 1.69) |
|  | Injury, poisoning and procedural complications | 74 | 1.92 (1.51 - 2.45) | 1.82 (28.81) | 1.81(1.48-2.22) | 0.86 (-0.81 - 2.53) |
|  | Cardiac disorders | 65 | 3.3 (2.55 - 4.27) | 3.07 (92.6) | 3.04(2.45-3.78) | 1.61 (-0.06 - 3.28) |
|  | Neoplasms benign, malignant and unspecified (incl cysts and polyps) | 47 | 1.53 (1.14 - 2.06) | 1.49 (7.96) | 1.49(1.16-1.91) | 0.57 (-1.1 - 2.24) |
|  | Blood and lymphatic system disorders | 41 | 0.53 (0.38 - 0.73) | 0.56 (16.13) | 0.56(0.43-0.73) | -0.84 (-2.51 - 0.83) |
|  | Investigations | 29 | 0.39 (0.27 - 0.57) | 0.42 (25.79) | 0.42(0.31-0.58) | -1.24 (-2.91 - 0.43) |
|  | Nervous system disorders | 22 | 0.87 (0.57 - 1.33) | 0.87 (0.43) | 0.87(0.61-1.25) | -0.2 (-1.87 - 1.47) |
|  | Gastrointestinal disorders | 20 | 0.42 (0.27 - 0.66) | 0.44 (15.48) | 0.44(0.3-0.64) | -1.19 (-2.86 - 0.48) |
|  | Hepatobiliary disorders | 18 | 1.48 (0.93 - 2.37) | 1.47 (2.72) | 1.47(0.99-2.17) | 0.55 (-1.12 - 2.22) |
|  | Skin and subcutaneous tissue disorders | 17 | 0.99 (0.61 - 1.61) | 0.99 (0) | 0.99(0.66-1.49) | -0.01 (-1.68 - 1.66) |
|  | Respiratory, thoracic and mediastinal disorders | 16 | 0.48 (0.29 - 0.78) | 0.49 (9.02) | 0.49(0.32-0.74) | -1.03 (-2.7 - 0.64) |
|  | Renal and urinary disorders | 16 | 1.38 (0.84 - 2.28) | 1.37 (1.65) | 1.37(0.9-2.08) | 0.46 (-1.21 - 2.13) |
|  | Pregnancy, puerperium and perinatal conditions | 8 | 9.97 (4.9 - 20.28) | 9.86 (61.42) | 9.53(5.26-17.27) | 3.25 (1.57 - 4.93) |
|  | Musculoskeletal and connective tissue disorders | 7 | 0.73 (0.35 - 1.54) | 0.73 (0.69) | 0.73(0.39-1.37) | -0.45 (-2.12 - 1.22) |
|  | Metabolism and nutrition disorders | 7 | 0.44 (0.21 - 0.92) | 0.44 (5.03) | 0.44(0.24-0.83) | -1.17 (-2.84 - 0.5) |
|  | Vascular disorders | 7 | 0.55 (0.26 - 1.17) | 0.56 (2.5) | 0.56(0.3-1.04) | -0.84 (-2.51 - 0.83) |
|  | Ear and labyrinth disorders | 2 | 2.66 (0.66 - 10.72) | 2.65 (2.04) | 2.63(0.82-8.47) | 1.4 (-0.28 - 3.08) |
|  | Eye disorders | 2 | 0.55 (0.14 - 2.2) | 0.55 (0.74) | 0.55(0.17-1.76) | -0.86 (-2.53 - 0.81) |
|  | Surgical and medical procedures | 1 | 0.07 (0.01 - 0.5) | 0.07 (12.27) | 0.07(0.01-0.37) | -3.79 (-5.47 - -2.12) |
|  | Psychiatric disorders | 1 | 0.15 (0.02 - 1.05) | 0.15 (4.93) | 0.15(0.03-0.77) | -2.75 (-4.42 - -1.08) |
|  | Immune system disorders | 1 | 0.12 (0.02 - 0.83) | 0.12 (6.72) | 0.12(0.02-0.61) | -3.09 (-4.76 - -1.41) |
| daunorubicin | Infections and infestations | 253 | 1.31 (1.14 - 1.5) | 1.25 (14.78) | 1.25(1.11-1.4) | 0.32 (-1.35 - 1.99) |
|  | Blood and lymphatic system disorders | 150 | 0.89 (0.75 - 1.06) | 0.9 (1.69) | 0.91(0.79-1.04) | -0.14 (-1.81 - 1.52) |
|  | Respiratory, thoracic and mediastinal disorders | 147 | 2.12 (1.79 - 2.52) | 2.01 (76.84) | 1.99(1.72-2.3) | 0.99 (-0.68 - 2.66) |
|  | Gastrointestinal disorders | 123 | 1.22 (1.01 - 1.46) | 1.2 (4.24) | 1.19(1.02-1.4) | 0.26 (-1.41 - 1.92) |
|  | Investigations | 123 | 0.77 (0.64 - 0.93) | 0.79 (7.57) | 0.79(0.68-0.93) | -0.34 (-2 - 1.33) |
|  | General disorders and administration site conditions | 117 | 0.52 (0.43 - 0.63) | 0.56 (47.67) | 0.56(0.48-0.66) | -0.84 (-2.5 - 0.83) |
|  | Cardiac disorders | 117 | 2.58 (2.13 - 3.13) | 2.45 (102.05) | 2.42(2.07-2.84) | 1.28 (-0.39 - 2.94) |
|  | Nervous system disorders | 80 | 1.43 (1.14 - 1.8) | 1.41 (9.72) | 1.4(1.16-1.7) | 0.49 (-1.18 - 2.16) |
|  | Vascular disorders | 65 | 2.38 (1.85 - 3.06) | 2.32 (48.86) | 2.29(1.86-2.83) | 1.2 (-0.47 - 2.87) |
|  | Metabolism and nutrition disorders | 42 | 1.18 (0.87 - 1.61) | 1.18 (1.16) | 1.18(0.91-1.52) | 0.24 (-1.43 - 1.9) |
|  | Injury, poisoning and procedural complications | 36 | 0.37 (0.27 - 0.52) | 0.39 (37.1) | 0.39(0.3-0.51) | -1.36 (-3.03 - 0.31) |
|  | Neoplasms benign, malignant and unspecified (incl cysts and polyps) | 33 | 0.45 (0.32 - 0.63) | 0.46 (21.9) | 0.46(0.35-0.62) | -1.11 (-2.78 - 0.55) |
|  | Skin and subcutaneous tissue disorders | 31 | 0.8 (0.56 - 1.14) | 0.8 (1.57) | 0.8(0.59-1.08) | -0.32 (-1.99 - 1.35) |
|  | Psychiatric disorders | 28 | 1.88 (1.29 - 2.73) | 1.86 (11.05) | 1.85(1.35-2.53) | 0.88 (-0.79 - 2.55) |
|  | Hepatobiliary disorders | 23 | 0.82 (0.54 - 1.25) | 0.83 (0.84) | 0.83(0.59-1.17) | -0.27 (-1.94 - 1.4) |
|  | Renal and urinary disorders | 22 | 0.83 (0.54 - 1.27) | 0.83 (0.74) | 0.83(0.59-1.19) | -0.26 (-1.93 - 1.41) |
|  | Musculoskeletal and connective tissue disorders | 13 | 0.6 (0.35 - 1.03) | 0.6 (3.49) | 0.6(0.38-0.95) | -0.73 (-2.4 - 0.94) |
|  | Pregnancy, puerperium and perinatal conditions | 10 | 5.51 (2.91 - 10.41) | 5.47 (34.96) | 5.27(3.09-8.98) | 2.4 (0.72 - 4.08) |
|  | Surgical and medical procedures | 10 | 0.31 (0.17 - 0.58) | 0.32 (15.02) | 0.32(0.19-0.54) | -1.65 (-3.32 - 0.02) |
|  | Immune system disorders | 5 | 0.26 (0.11 - 0.62) | 0.26 (10.72) | 0.26(0.12-0.54) | -1.94 (-3.61 - -0.27) |
|  | Eye disorders | 5 | 0.61 (0.25 - 1.46) | 0.61 (1.27) | 0.61(0.29-1.27) | -0.71 (-2.38 - 0.96) |
|  | Reproductive system and breast disorders | 3 | 1.34 (0.43 - 4.2) | 1.34 (0.26) | 1.34(0.52-3.47) | 0.42 (-1.26 - 2.1) |
|  | Congenital, familial and genetic disorders | 1 | 0.43 (0.06 - 3.06) | 0.43 (0.76) | 0.43(0.08-2.23) | -1.21 (-2.89 - 0.46) |
|  | Endocrine disorders | 1 | 0.5 (0.07 - 3.53) | 0.5 (0.51) | 0.5(0.1-2.58) | -1.01 (-2.68 - 0.67) |

Reference:

1. Dai, Z., et al., *Adverse events associated with eteplirsen: A disproportionality analysis using the 2016-2023 FAERS data.* Heliyon, 2024. **10**(13): p. e33417.

2. Lin, G., et al., *Analyzing real-world adverse events of spironolactone with the FAERS database.* PLoS One, 2025. **20**(9): p. e0330659.
